# Validation of next-generation sequencing-based chimerism testing for accurate detection and monitoring of engraftment in hematopoietic stem cell transplantation

**DOI:** 10.3389/fgene.2023.1282947

**Published:** 2023-10-23

**Authors:** Pramath Kakodkar, Yayuan Zhao, Henry Pan, Fang Wu, Twyla Pearce, Destinie Webster, Mohamed Elemary, Waleed Sabry, Luvinia Kwan, Lindsay Pelzer, Mark Bosch, Karen R. Sherwood, James Lan, Jenny Tran, Robert Liwski, Paul Keown, Ahmed Mostafa

**Affiliations:** ^1^ Department of Pathology and Laboratory Medicine, University of Saskatchewan, Saskatoon, SK, Canada; ^2^ Histocompatibility and Immunogenetics Laboratory, St. Paul’s Hospital, Saskatoon, SK, Canada; ^3^ Department of Hematological Oncology, Saskatchewan Cancer Agency, Saskatoon, SK, Canada; ^4^ HLA Laboratory, Cancer Care Manitoba, Winnipeg, MB, Canada; ^5^ University of British Columbia, Vancouver Coastal Health, Vancouver, BC, Canada; ^6^ Department of Transplant Nephrology, Vancouver Coastal Health, Vancouver, BC, Canada; ^7^ Department of Pathology, Dalhousie University, Halifax, NS, Canada

**Keywords:** next-generation sequencing, chimerism, cell subsets, standardization, proficiency testing

## Abstract

Allogenic hematopoietic stem cell transplantation (allo-HSCT) is a life-saving treatment for various hematological disorders. The success of allo-HSCT depends on the engraftment of donor cells and the elimination of recipient cells monitored through chimerism testing. We aimed to validate a next-generation sequencing (NGS)-based chimerism assay for engraftment monitoring and to emphasize the importance of including the most prevalent cell subsets in proficiency testing (PT) programs. We evaluated the analytical performance of NGS-based chimerism testing (AlloSeq-HCT and CareDx) with a panel of targeted 202 informative single-nucleotide polymorphisms (SNPs) (i.e., linearity and precision, analytical sensitivity and specificity, system accuracy, and reproducibility). We further compared the performance of our NGS panel with conventional short tandem repeat (STR) analysis in unfractionated whole blood and cell-subset-enriched CD3 and CD66. Our NGS-based chimerism monitoring assay has an impressive detection limit (0.3% host DNA) for minor alleles and analytical specificity (99.9%). Pearson’s correlation between NGS- and STR-based chimerism monitoring showed a linear relationship with a slope of 0.8 and r = 0.973. The concordance of allo-HSCT patients using unfractionated whole blood, CD3, and CD66 was 0.95, 0.96, and 0.54, respectively. Utilization of CD3^+^ cell subsets for mixed chimerism detection yielded an average of 7.3 ± 7-fold higher donor percentage detection compared to their corresponding unfractionated whole blood samples. The accuracy of the NGS assay achieved a concordance of 98.6% on blinded external quality control STR samples. The reproducibility series showed near 100% concordance with respect to inter-assay, inter-tech, inter-instrument, cell flow kits, and AlloSeq-HCT software versions. Our study provided robust validation of NGS-based chimerism testing for accurate detection and monitoring of engraftment in allo-HSCT patients. By incorporating the cell subsets (CD3 and CD66), the sensitivity and accuracy of engraftment monitoring are significantly improved, making them an essential component of any PT program. Furthermore, the implementation of NGS-based chimerism testing shows potential to streamline high-volume transplant services and improve clinical outcomes by enabling early relapse detection and guiding timely interventions.

## 1 Introduction

Allogeneic hematopoietic stem cell transplantation (allo-HSCT) is a form of curative treatment for a variety of hematological malignancies, such as acute leukemias, lymphomas, myelodysplastic syndromes, plasma cell disorders, myeloproliferative neoplasms, and other genetic disorders. The annual allo-HSCT rate in Canada from 2008 to 2019 has been 926 ± 107 transplants (CTTC. Cell, 2021). This prevalence stems from the impact of allo-HSCT in concatenating the positive rates for remission and overall survival in both pediatric and adult cohorts (Svenberg et al., 2016; Appelbaum, 2017; Döhner et al., 2017). Despite this success, allo-HSCT can cause a myriad of complications, such as treatment-associated toxicity, relapse, graft-versus-host disease (GvHD), and death. Therefore, clinicians need to utilize newer tools post-allo-HSCT to monitor complications, minimal residual disease status, and propensity for rejection.

The success of allo-HSCT is measured by engraftment, where the donor cells initiate the production of healthy hematopoietic stem cells against a background of complete eradication of pre-transplant hematological/hemato-oncological disorders. Engraftment at a cellular level can be substantiated by chimerism, which refers to the ratio of the genetically distinct donor and recipient cell populations. The delineation of the ratio of these cell populations is dependent on factors such as the intensity of the conditioning regimen, GvHD prophylaxis, the recipient’s prior chemotherapy regime, and graft composition. Mixed chimerism is defined as either the persistence or relapse of the host non-neoplastic cells or, in the worst-case scenario, the re-emergence or repopulation of the neoplastic cells. Allo-HSCT patients with mixed chimerism (MC) show an increased proclivity for graft rejection and disease recurrence (Busque et al., 2020). Chimerism testing can be instrumental in the longitudinal monitoring of the patient’s immune convalescence and cellular reconstitution post-allo-HSCT, monitoring engraftment kinetics and trends in donor engraftment in the follow-up period.

Next-generation sequencing (NGS) has emerged as a promising tool for chimerism monitoring due to its high sensitivity, accuracy, and multiplexing capacity. In comparison to already established methods such as short tandem repeats (STRs), quantitative polymerase chain reaction (qPCR), or fluorescence *in situ* hybridization (FISH), NGS reigns supreme in the detection of minuscule fractions of donor cells within the recipient’s blood or bone marrow. Chimerism monitoring with NGS depends on either sequencing informative single-nucleotide polymorphisms (SNPs) or InDel panels from the donor and recipient DNA samples. These results are then analyzed via specialized software to quantify the relative ratio of donor and recipient alleles. NGS allows for the detection of low-level chimerism with a sensitivity of ∼0.1–0.5%, compared to ∼1–5% with these conventional methods (Blouin and Askar, 2022). This could be useful in predicting graft failure after full engraftment and early detection of potential complications, influencing clinical decision-making, and improving patient outcomes in allo-HSCT and other cellular therapies. A previous study reported that NGS-based chimerism monitoring could predict relapse with high accuracy across adult patients (n = 75) undergoing allo-HSCT with low, intermediate, and high MC (Pettersson et al., 2021).

Cell subset analysis plays a crucial role in NGS-based chimerism testing. Isolating specific cell subsets such as CD3-positive T lymphocytes, CD19-positive B cells, and CD66-positive myeloid cells provides more accurate representation of the post-allo-HSCT dynamics than a whole blood analysis (Lion et al., 2012). Distinct chimerism patterns can be gleaned, and these can provide a comprehensive understanding of the immune reconstitution process or temporization of engraftment failure.

The current literature reveals a gap in incorporating lineage-specific cell subset-based chimerism analysis into proficiency testing. The large-scale adaptation of PT will enable laboratories to assess their proficiency metrics, such as accuracy, consistency, reliability, and comparability of results across different laboratories. Incorporating these quality assurances will result in accurate and standardized assessments of engraftment outcomes via cell subset testing, potentially revolutionizing and facilitating clinical decision-making.

In this study, we validated the NGS-based assay with a panel of the target 202 loci of known biallelic SNPs. These biallelic SNPs were selected from the 1000 Genomes Project due to their high heterozygosity and lack of linkage disequilibrium, ensuring that each SNP provides independent information about chimerism (Zhang et al., 2015). After sequencing, the resulting data were analyzed to determine the percentage of donor and recipient alleles at each SNP locus. The analytical performance and clinical utility of NGS-based chimerism monitoring were summarized.

## 2 Methods

### 2.1 Samples

A total of 196 post-transplant samples and 54 genomic samples were subjected to analysis using the NGS-based chimerism assay (CareDx, Stockholm, Sweden), and the results were compared with those from the STR assay (AmpFLSTR™ Identifiler™ Plus PCR Amplification Kit, Applied Biosystems). These samples were obtained from 27 patient/donor pairs. Among them, 22 pairs were obtained from allo-HSCT cases, while the remaining 32 pairs were obtained from unrelated allo-HSCT cases. Prior to method validation, approval was obtained from the Saskatchewan Cancer Agency (SCA) Privacy Office to utilize de-identified residual samples for method validation and scientific research.

As part of the external quality control (EQC), we received 22 blind post-transplant samples and 10 genomic samples from HLA Laboratory, Cancer Care Manitoba, Winnipeg, MB, Canada. Samples were obtained as whole blood (n = 13) and bone marrow (n = 3). Enriched samples were obtained as CD3-positive T cells (n = 2), CD19-positive neoplastic B cells (n = 2), and CD66-positive myeloid cells (n = 2). In addition, 15 whole blood post-transplant samples and six genomic samples were supplied as part of the American Society of Histocompatibility and Immunogenetics (ASHI) proficiency testing program.

### 2.2 Cell enrichments

To increase the assay sensitivity for minor cell fractions, cell enrichment was conducted from the whole blood prior to DNA extraction using the EasySep™ Human Whole Blood Positive Selection Kit (STEMCELL Technologies, Vancouver, Canada) for CD3-positive T cells (Catalog #18081) and CD66b/33-positive myeloid cells (Catalog #18683), followed by DNA extraction, according to manufacturer’s recommendation. Cells were eluted in 300 μL of EasySep buffer (STEMCELL Technologies, Vancouver, Canada) and then counted using the Countess cell counter (Thermo Fisher Scientific, Canada).

### 2.3 DNA extraction

DNA extraction was performed, as previously described by Kakodkar et al. (2023). Briefly, DNA extractions were prepared by QIAGEN, using the BioRobot^®^ EZ1 system (QIAGEN, Toronto, Canada) and EZ1 DNA Blood 350 μL Kit (Catalog 951,054), using whole blood collected in acid citrate dextrose tubes and isolated cell fractions. The DNA concentration and purity were quantified using a NanoDrop spectrophotometer (Thermo Fisher Scientific, Canada), and samples with a 260/280 ratio >1.8 were processed. Extracted DNA samples were normalized to 0.625 ng/μL using PCR-grade water (Thermo Fisher Scientific, Canada) to fulfill the 10-ng input requirement in a 16 μL volume. DNA was stored at a temperature range of 2°C to 8°C for up to 1 week and was subsequently frozen (−20°C).

### 2.4 NGS chimerism assay

The targeted NGS-based assay (AlloSeq HCT) was performed in accordance with the manufacturer’s instructions (CareDx, Stockholm, Sweden). Briefly, one PCR amplification cycle was performed, using the target DNA (0.625 ng/μL), PCR master mix (PCR Mix, SNP primer pool, and PCR enzyme), dual sample-specific indices, and flow-cell adapters. Following the PCR amplification, the products were pooled and cleaned using the AlloSeq HCT purification beads. The final library concentration was measured using the Qubit Flex Fluorometer (Thermo Fisher Scientific, Canada), followed by dilution and denaturation using 2 N NaOH (supplied in the kit). The final library was diluted to 2 pm and spiked with 1% PhiX (Illumina, Canada). Depending on the total number of samples, the final library was loaded into either the mid-output (Illumina, Canada Cat# FC-420-1001) or the high-output (Illumina, Canada Cat# FC-420-1002) flow cell and sequenced on the MiniSeq instruments (Illumina, Canada). FASTQ files generated using MiniSeq were imported into AlloSeq HCT software versions 1 and 2.1.2 (CareDx, Stockholm, Sweden).

### 2.5 Short tandem repeat assay

The STR assay was performed at HLA Laboratory, Cancer Care Manitoba, Winnipeg, Canada, according to the manufacturer’s recommendation (AmpFLSTR™ Identifiler™ Plus PCR Amplification Kit, Applied Biosystems). The STR assay was performed on a 3500xL Genetic Analyzer (Applied Biosystems, Foster City, CA) using one injection per sample (four-color, 16-plex detection). Analysis was conducted on GeneMapper v.4.1 (Applied Biosystems), and the corresponding electropherograms were printed for each sample and interpreted manually.

## 3 Results

### 3.1 Linearity and precision

To validate linearity and precision, seven artificial DNA mixtures were created. These mixtures were prepared by diluting DNA samples within the range of 0.3%–50%, adhering to the predetermined proportions outlined in Supplementary Table S1. The primary objective was to maintain precise and accurate measurements throughout the experiment. Each of these samples underwent triplicate runs utilizing the AlloSeq HCT Kit. Subsequently, they were sequenced using the MiniSeq instruments (Illumina, Canada).

**FIGURE 1:**
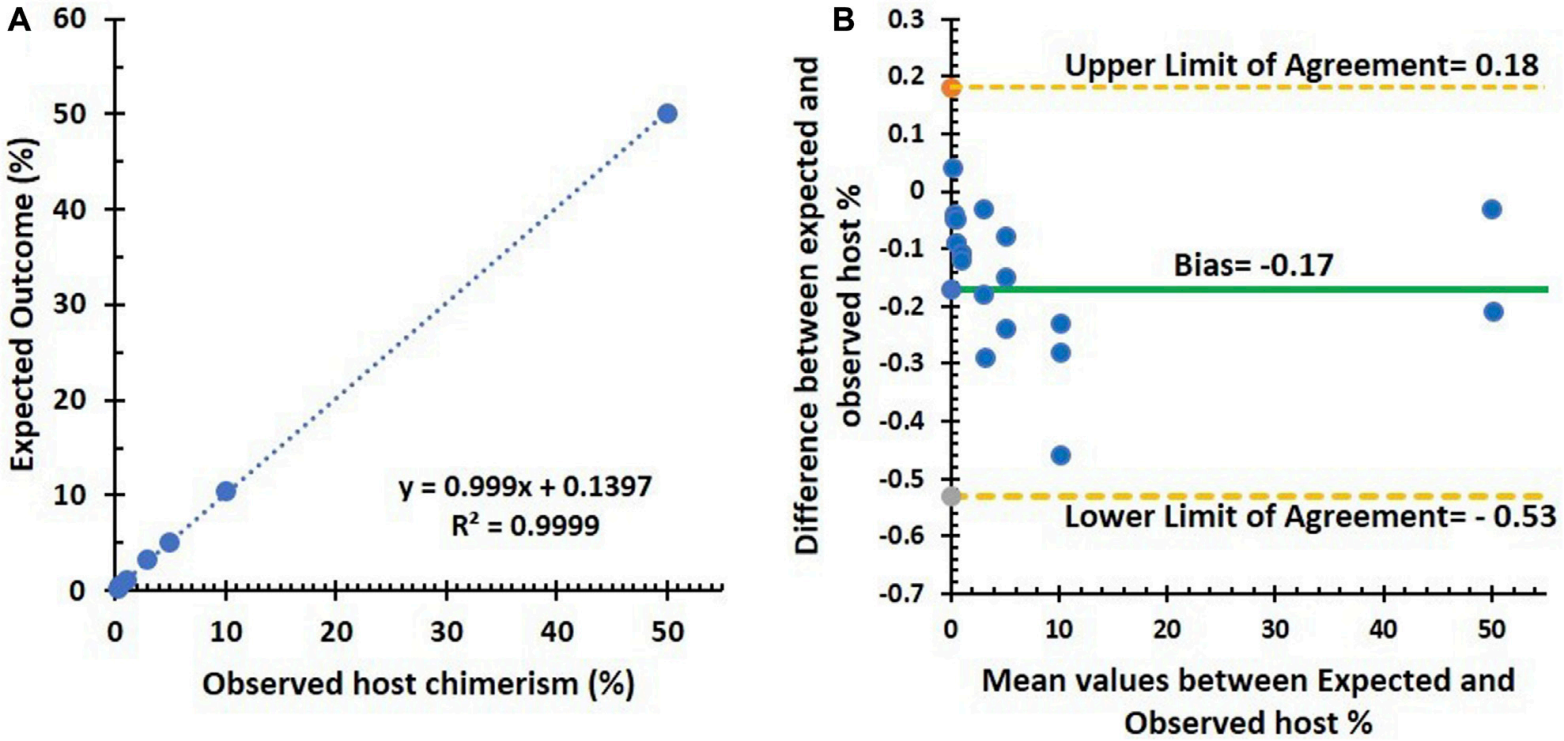
Summary of linearity and precision testing results. **(A)** Correlation (Pearson’s correlation coefficient = 0.99, *p* < 0.001) between observed and expected host % using the NGS assay in seven artificial DNA samples diluted from 0.3% to 50%. **(B)** Bland–Altman plot between observed and expected host %: an average difference of −0.17% host chimerism (green line), with 95% limits of agreement [−0.53 to 0.18] (dashed lines).

There was a strong linear relationship between the observed and expected outcomes, with a Pearson correlation coefficient of 0.99 (*p* < 0.001). The standard deviation (SD) ranges from 0.3 to 0.004, indicating a high level of precision. This precision is further supported by fixed SD among the replicates, as shown in Figure 1A. Additionally, Figure 1B, the Bland–Altman plot, demonstrates the assay’s high precision, with the triplicates tightly clustered across the dilution series.

### 3.2 Analytical sensitivity and specificity

**FIGURE 2:**
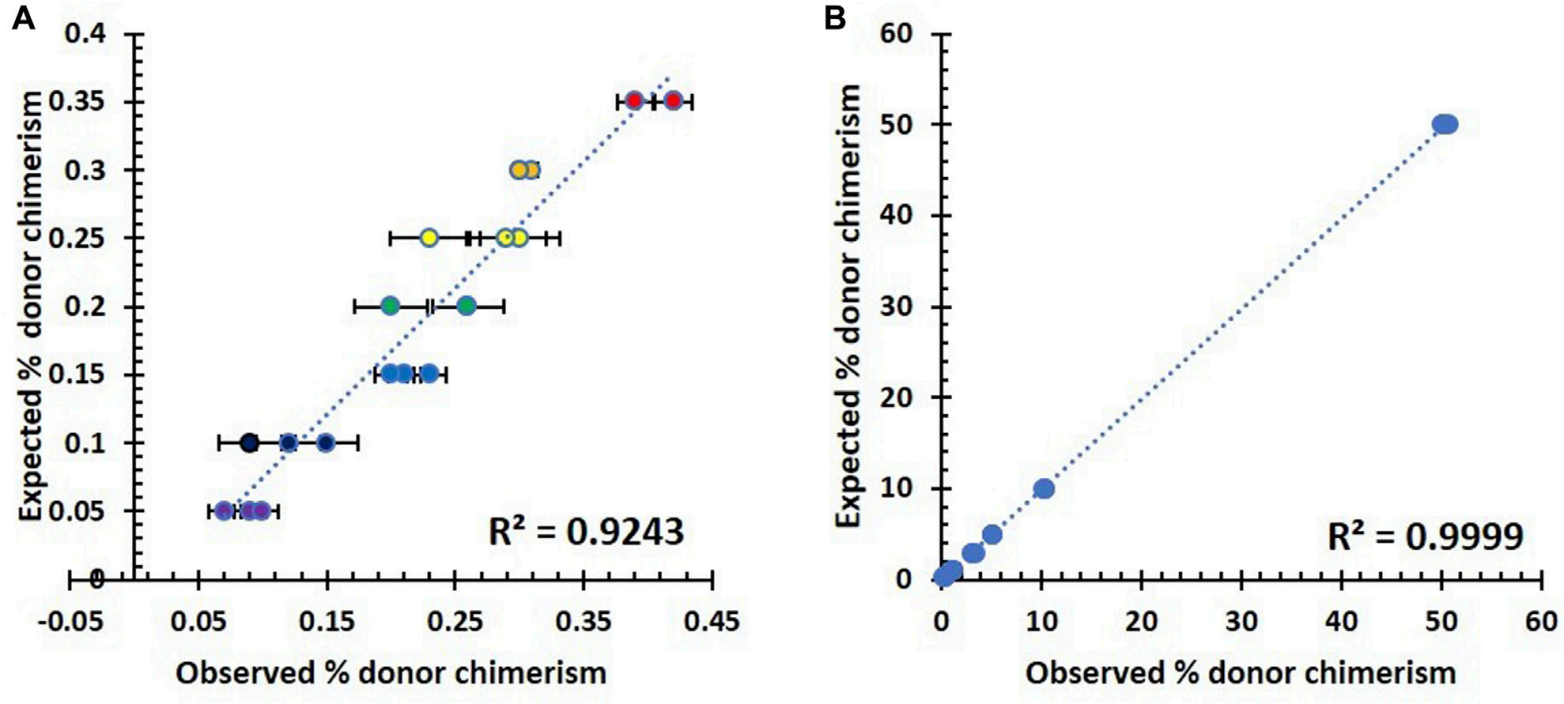
Summary of analytical sensitivity testing in seven triplicates of artificial DNA samples. **(A)** Correlation between observed and expected % host chimerism using the NGS assay in the lower fraction (0.05–0.35%). **(B)** Correlation between observed and expected % host chimerism using the NGS assay in the higher fraction (0.3–50%).

In order to simplify the analysis, we created two graphs to compare the observed donor DNA percentages with the expected values. Figure 2A represents the results for the lower fractions of the chimerism mixture, which range from 0.05% to 0.35%. Figure 2B, on the other hand, shows the corresponding results for the chimerism mixture, with the higher fractions ranging from 0.3% to 50%.

As shown in Figure 2, there was a direct proportional relationship between the observed and expected percentage of host chimerism by the NGS assay across the lower (Figure 2A) and higher fractions (Figure 2B) of mixed chimerism samples. The lower fraction has a slightly higher variation within its repeats, and the resultant correlation is 0.9243. Contrastingly, the higher fraction showed a constrained variation with a significantly higher correlation of 0.9999. The accurate measurement of the 0.3% minority fraction proved to be reproducible (standard deviation 0.005). Therefore, the lowest detection limit for our NGS assay was chosen to be 0.3%.

To determine analytical specificity, we utilized two distinct DNA samples, and these were further split into two aliquots. One aliquot from each DNA sample was intentionally labeled as Recipient (Ref1) and Donor (Ref2) to serve as genomic reference DNA. The remaining two aliquots were labeled as Recipient (post-transplant) and Donor (post-transplant), representing complete host or complete donor chimerism after the transplant (Supplementary Table S2). All DNA samples were processed using the AlloSeq HCT Kit and subsequently sequenced using the MiniSeq instruments (Illumina, Canada). The background noise levels detected in Ref1 and Ref2 were 0.05% and 0.06%, respectively. Additionally, the measured background noise levels in Recipient (post-transplant) and Donor (post-transplant) were 0.05% and 0.04%, respectively. By comparing the specificity of the post-transplant samples with their respective reference genomic DNA, we concluded that the background signal in our NGS assay ranged from 0.04% to 0.06%. We approximated the background signal to be 0.1%. Therefore, the specificity of our NGS assay is 99.9%.

### 3.3 Limit of the sample input

Two duplicates were prepared for each DNA mixture using a serial dilution of DNA ranging from 10 ng to 1.25 ng. An artificial chimerism mixture was prepared from each DNA concentration for an expected concentration of 100% (neat); 15% and 85% (mixture 1); and 50% and 50% (mixture 2) and run on the NGS assay. The overall summary of the aforementioned mixture schema and coefficients of variance are shown in Table 1. The concordance between the observed and expected DNA concentrations was recorded. The coefficients of variance (CV) for the neat, mixture 1, and mixture 2 groups were 0.013%, 0.69%, and 0.53% respectively. These miniscule CVs indicate a near homogenous dataset, and the comparisons between the expected and observed DNA input are repeatable and can detect DNA fractions in DNA sample inputs as low as 1.25 ng.

**TABLE 1:** Summary of two replicates of the four DNA samples. Expected (%) and observed (%) DNA output from the four input DNA samples (1.25 ng, 2.5 ng, 5 ng, and 10 ng) and their respective mixtures: neat (100%), mixture 1 (15%: 85%), and mixture 2 (50%:50%). Data variability between the expected (%) and observed (%) DNA output is measured with the coefficient of variance statistical test.

	Sample input	Replicate	Neat		Mixture 1		Mixture 2	
Expected output (%)			100%		15% and 85%		50% and 50%	
**Observed output (%)**	**10 ng DNA**	**Replicate 1**	0.08	99.92	14.4	85.6	49.33	50.67
		**Replicate 2**	0.07	99.93	14.6	85.41	49.37	50.63
	**5 ng DNA**	**Replicate 1**	0.09	99.91	14.1	85.87	49.74	50.26
		**Replicate 2**	0.06	99.94	15.1	84.87	49.36	50.64
	**2.5 ng DNA**	**Replicate 1**	0.07	99.93	13.6	86.37	49.03	50.97
		**Replicate 2**	0.06	99.94	14.7	85.13	49.92	50.08
	**1.25 ng DNA**	**Replicate 1**	0.05	99.95	13.5	86.51	49.27	50.73
		**Replicate 2**	0.05	99.95	13.5	86.51	49.27	50.73
	**Mean**			99.93375	14.19375	85.78375	49.41125	50.58875
	**Standard deviation**			0.01317	0.573889	0.596006	0.265303	0.265303
	**Coefficient of variance**			0.013178	4.043251	0.694778	0.536929	0.524432

The utilization of bold titles serves to enhance the differentiation between the section headings and the presented results.

### 3.4 Reproducibility

**FIGURE 3:**
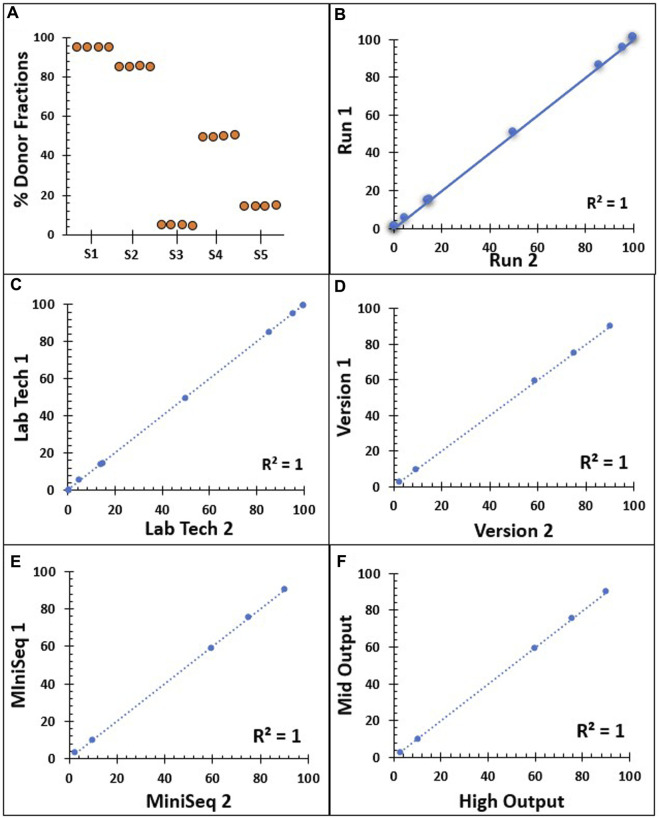
Reproducibility series summarizing the NGS assay donor % results. **(A)** Inter-assay reproducibility on the same run, conducted on four repeats with five different donor % samples (S1–S5) by a single technician. **(B)** Inter-assay reproducibility on two different runs on 11 samples with different donor % samples by a single technician. **(C)** Inter-tech reproducibility by two different laboratory technologists on eight duplicate samples. **(D)** Output from five different donor % samples analyzed using Alloseq-HCT software versions 1 and 2.1. **(E)** Correlation between two NSG instruments, Illumina MiniSeq1 and MiniSeq2 on five different donor % samples. **(F)** Reproducibility comparison between mid-output and high-output cell flow kits on 14 duplicate samples from different donor % samples. All dashed and solid lines in B–F indicate the trend lines, and all five Pearson’s correlation coefficient values are 0.999.

Inter-assay reproducibility was performed on four replicates of five different samples, where a single NGS assay was performed by the same technologist on the same run for each sample (Figure 3A). Similarly, 11 samples were tested in duplicate by the same technologist on two different runs (Figure 3B). The reproducibility of our NGS-based MC monitoring assay showed a concordance of almost 100% when testing the same samples four times in the same run and when repeated in separate runs. To observe the concordance in inter-tech variance, eight samples were tested in duplicate by two different technologists on a different run (Figure 3C). Similarly, the output from five samples was analyzed using Alloseq-HCT software versions 1 and 2.1 (Figure 3D). The concordance for the donor % with both the two laboratory technologists and the two different software versions was 99.99%. Moreover, five samples were tested in duplicate by the same technologist on a different instrument to validate the inter-instrumental variance in reporting the donor % between Illumina MiniSeq1 and MiniSeq2 (Figure 3E). Similarly, 14 samples were tested in duplicate to compare the variance in detecting donor % between the Illumina mid-output and the high-output cell flow kits (Figure 3F). The concordance for these comparisons remained near 100%. The overall findings from the reproducibility showed direct proportional comparative outcomes, with all the aforementioned variables highlighting 99.99% concordance throughout.

### 3.5 Method comparison (STR vs. NGS assay)

**FIGURE 4:**
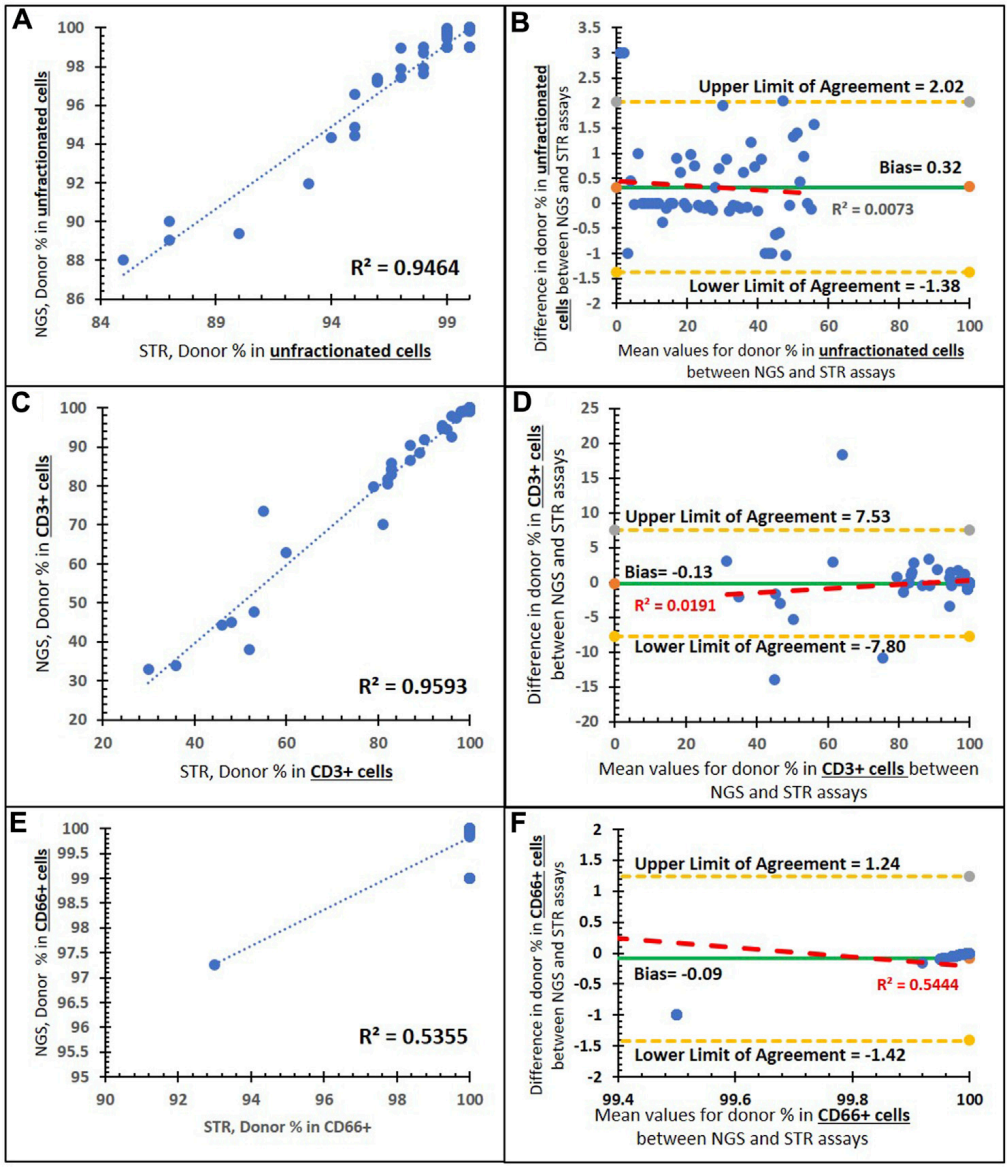
Method comparison of the NGS and STR assays. **(A)** Correlation between the two assays in unfractionated cells with a trend line (dashed blue line), an R^2^ value of 0.9464, and a Pearson’s correlation coefficient of 0.973 (*p* < 0.001). **(B)** Bland–Altman plot of the two assays in unfractionated cells: an average difference of 0.32% host chimerism (green solid line) is observed, with 95% limits of agreement [-1.38 to 2.02] (yellow dashed lines). **(C)** Correlation between the two assays in CD3^+^-enriched cells with a trend line (blue dashed), an R^2^ value of 0.959,3 and a Pearson’s correlation coefficient of 0.979 (*p* < 0.001). **(D)** Bland–Altman plot of the two assays in CD3^+^-enriched cells: an average difference of −0.13% host chimerism (green solid line) is observed, with 95% limits of agreement [−7.80 to 7.53] (yellow dashed lines). **(E)** Correlation between the two assays in CD66^+^-enriched cells with a trend line (blue dashed), an R^2^ value of 0.535, and a Pearson’s correlation coefficient of 0.73 (*p* < 0.001). **(F)** Bland–Altman plot of the two assays in CD66^+^-enriched cells: an average difference of −0.09% host chimerism (green solid line) is observed, with 95% limits of agreement [−1.42 to 1.42] (yellow dashed lines).

**FIGURE 5:**
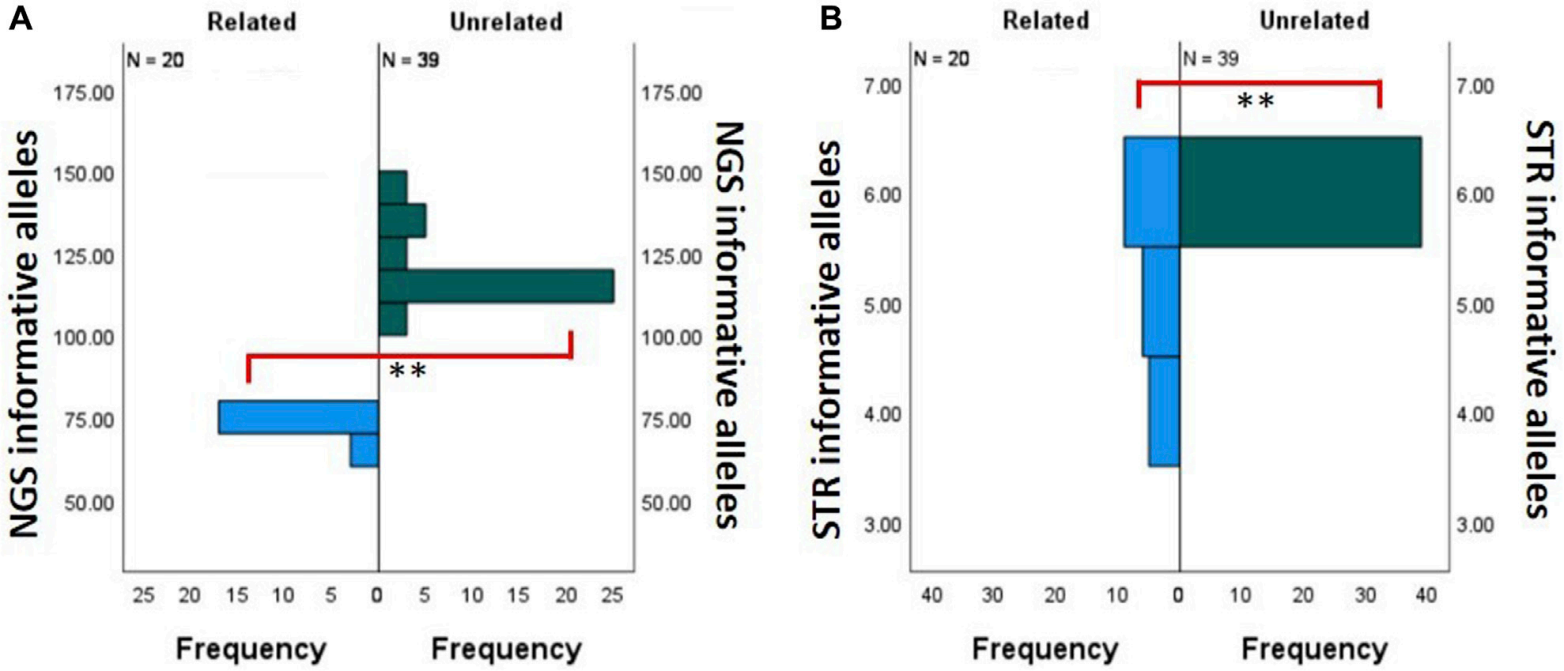
Comparison of the number of informative alleles. Related (n = 20, blue) and unrelated (n = 39, green) donor–recipient pairs. **(A)** Comparison bar graph for the NGS assay. **(B)** Comparison bar graph for the STR assay. ** Statistically significant with Mann–Whitney *U* test with *p* < 0.001.

To detect the accuracy of the NGS assay, we used 196 post-transplant clinical samples that were previously analyzed using the STR assay, as our parallel sample testing. Figure 5 shows the comparison of % donor chimerism between our NGS chimerism assay and parallel sample testing (STR assay) There is a positive linear correlation between the NGS and STR assays in unfractionated (Figure 4A), CD3^+^ (Figure 4C), and CD66^+^ cells (Figure 4E). Pearson’s correlation was higher in the comparison of % donor chimerism between the NGS and STR assays within unfractionated cells (0.973) and CD3^+^ cells (0.979) relative to the CD66^+^ (0.73) input sample. The Bland–Altman plot shows that the bias line is near 0 for unfractionated (0.32) (Figure 4B), CD3^+^ (−0.13) (Figure 4D), and CD66^+^ cells (−0.09) (Figure 4F), which indicates a high level of agreement between the NGS and STR assay methods. Similarly, the Bland–Altman plot shows that a low *R* ^2^ value for unfractionated cells (0.007) (Figure 5B) and CD3^+^ cells (0.019) (Figure 4D) indicates a lack of systematic bias. Contrastingly, CD66^+^ shows an *R* ^2^ value of 0.54 (Figure 4F), which indicates some minor underlying systematic bias.

Due to the limited number of overall markers in the STR-based MC assay, it is inherently restrictive to find multiple informative loci between donor and recipient pairs. This phenomenon is exaggerated when these are related donor–recipient pairs. Therefore, we compared the number of informative markers utilized between related and unrelated donor–recipient pairs’ runs on our NGS-based MC assay and the corresponding STR-based MC assay. Figure 5A shows the difference between the frequency distribution of NGS informative alleles in related (n = 22) and unrelated (n = 32) donors. In the NGS assay, there was a statistically significant difference (Mann–Whitney *U* test, *p* < 0.001) between the mean frequency of informative alleles within the unrelated (120.10 ± 1.61) and related (72.35 ± 2.45) donors. Figure 5B shows the difference between the frequency distribution of STR informative alleles in related (n = 20) and unrelated (n = 39) donors. In the STR assay, there was a statistically significant difference (Mann–Whitney *U* test, *p* < 0.001) between the mean frequency of informative alleles within the unrelated (6 ± 0.0) and related (5.2 ± 0.19) donors. In conclusion, although the number of informative loci in both comparisons was significant (*p* < 0.001), we observe that the STR mean difference is only 0.8, whereas in NGS, it is much larger at 47.75. The overall summary of the independent sample Mann–Whitney *U* test for related and unrelated donor populations in NGS- and STR-based MC monitoring is shown in Supplementary Table S3.

### 3.6 Comparison between unfractionated blood and CD3^+^-enriched input in the NGS assay

**FIGURE 6:**
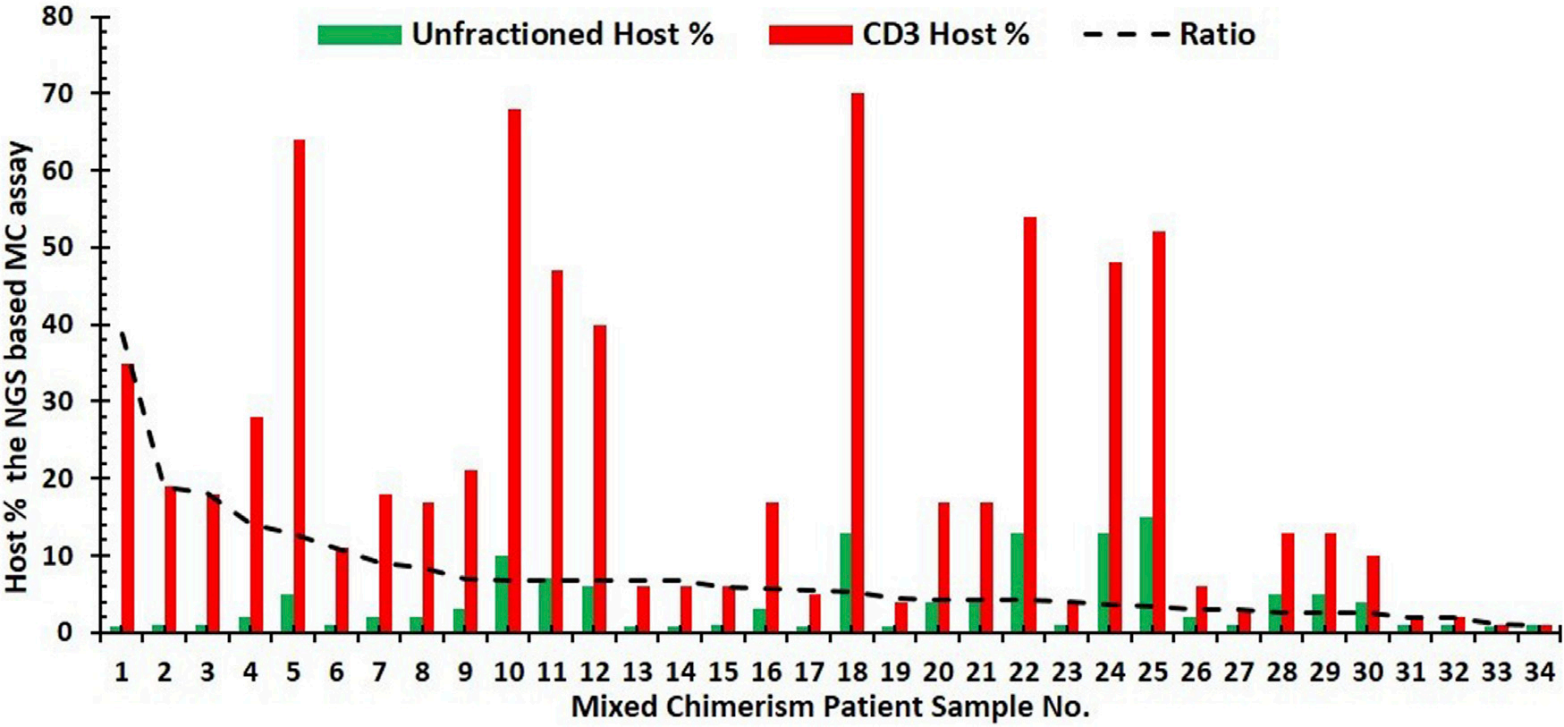
Bar graph of NGS results for the mixed chimerism case. Patient’s (n = 34) represented by the host % for unfractionated (green) and corresponding CD3-enriched (red) input samples. Trend line (dashed black line) for the ratio of host% in CD3-enriched cells to that of the unfractionated cells.

To identify the significance of cell subsets on NGS-based mixed chimerism monitoring, the concordance and intensity of mixed chimerism in the CD3-enriched cells were compared to the unfractionated blood samples. Mixed chimerism was identified in 36 patient samples. Of these cases of mixed chimerism, 94.4% (n = 34/36) had a concurrent increase in host % chimerism. The remaining two samples showed either a CD3^+^ (n = 1/36) increase without any unfractionated increase or an unfractionated increase (n = 1/36) without any CD3^+^ increase. Figure 6 shows NGS results for mixed chimerism represented as host % for unfractionated and CD3-enriched input samples. Mixed chimerism was detected with a higher intensity on CD3-enriched cells compared to unfractionated cells, as shown by the mean 7.1 ± 7.0-fold higher host % detected on CD3-enriched cells compared to the unfractionated samples. This fold change of host % for CD3-enriched to unfractionated input samples ranged from 38.9 to 1.

### 3.7 Comparison between the NGS assay and external quality control

Twenty-two blind samples were utilized to compare the donor % results between our laboratory’s NGS assay and the STR assay from Manitoba provincial HLA laboratory. Similarly, we compared 15 blind samples between our laboratory’s NGS assay to the ASHI PT samples, including 65 participated laboratories.

**FIGURE 7:**
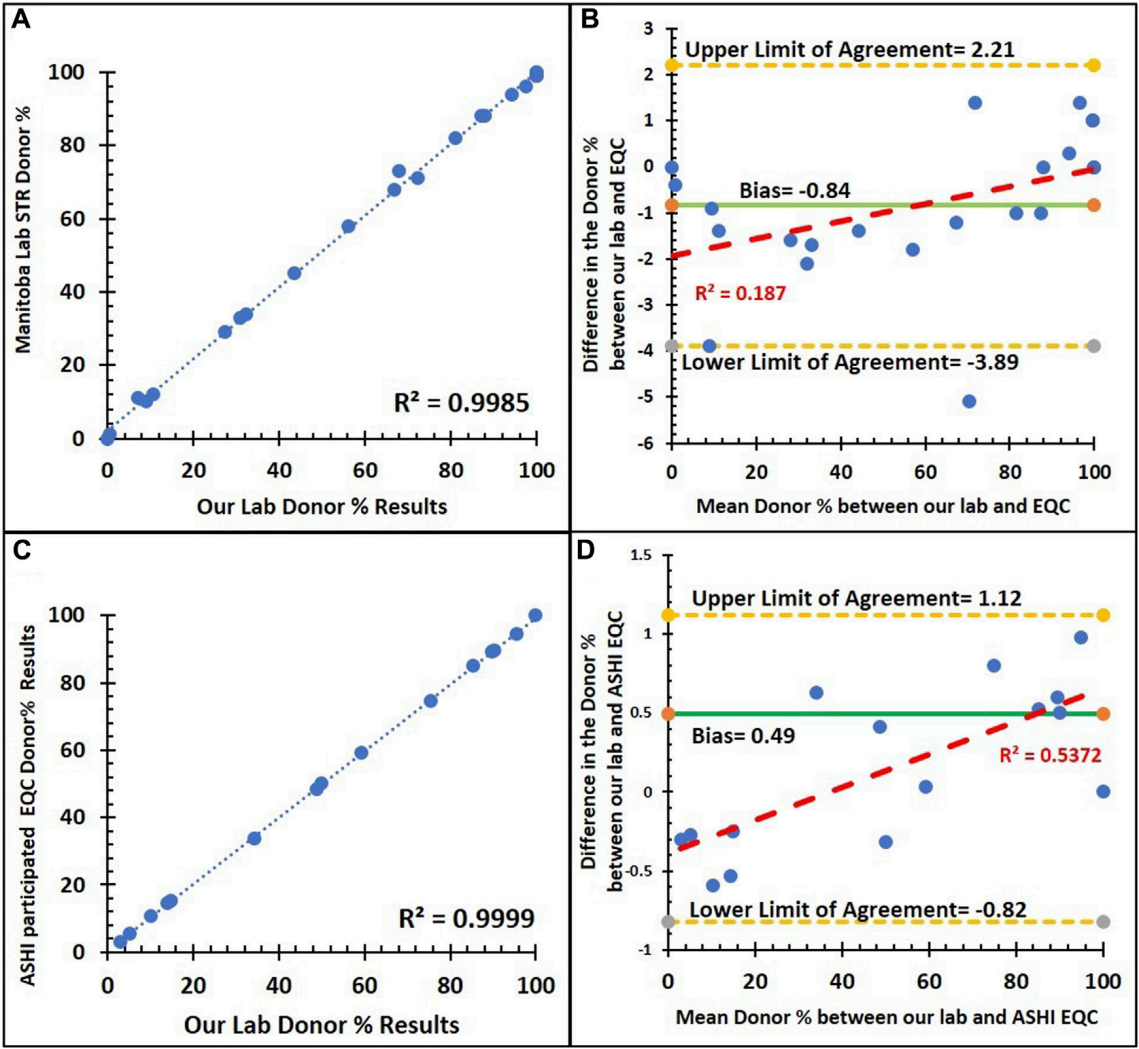
Method comparison of our institution NGS assay and external quality control with the STR assay. **(A)** Correlation between the donor % of the NGS and STR assays from the Manitoba provincial HLA laboratory (n = 22) with a trend line (dashed blue line), an R^2^ value of 0.9985, and a Pearson’s correlation coefficient of 0.999 (*p* < 0.001). **(B)** Bland–Altman plot for donor % between the NGS and STR assays from the Manitoba provincial HLA laboratory: an average difference of −0.84% host chimerism (green solid line) is observed, with 95% limits of agreement [−3.89 to 2.21] (yellow dashed lines). **(C)** Correlation between the donor % of NGS and STR assays from ASHI PT samples (n = 15) with a trend line (dashed blue line), an R^2^ value of 0.9999, and a Pearson’s correlation coefficient of 0.999 (*p* < 0.001). **(D)** Bland–Altman plot for donor % between the NGS and STR assays from ASHI PT samples: an average difference of 0.49% host chimerism (green solid line) is observed, with 95% limits of agreement [−0.82 to 1.12] (yellow dashed lines).

As shown in Figure 7, the comparison of % donor chimerism between our NGS chimerism assay and blind samples that was previously analyzed by STR assay by the Manitoba provincial HLA laboratory (n = 22) and ASHI Proficiency Testing samples (EMO) (n = 15). There is a positive linear correlation between our NGS and both blind samples. The Pearson correlation is nearly 100%. Figures 7B, D show the Bland–Altman plots with their bias line at near 0 for comparisons of % donor chimerism between STR and EQC for the Manitoba HLA laboratory (−0.84%) and ASHI participated laboratories (0.49%), which indicates a high level of agreement between NGS and the PT samples. Similarly, the Bland–Altman plot shows that the low *R* ^2^ value for the Manitoba HLA laboratory (0.187) indicates the lack of systematic bias, and moderate *R* ^2^ for ASHI-participated laboratories (0.537), which indicates some minor underlying systematic bias. Supplementary Table S4 shows that the mean donor % of the STR based MC assay from the ASHI PT samples compared to our NGS-based MC monitoring assay were consistently around the mean donor % of the ASHI PT.

## 4 Discussion

Chimerism monitoring remains instrumental in the management of post-allo-HSCT patients by detecting the status of engraftment, early graft failure, and disease relapse. We are the first Canadian clinical laboratory to validate and implement NGS-based cell subset chimerism monitoring in allo-HSCT patients. The guidance in the scientific literature for PT in NGS-based chimerism testing with lineage-specific cell-subsets remains nearly non-existent. We have, therefore, included our comparative inter-laboratory lineage-specific cell subset chimerism testing as a proxy for PT in the absence of commercially available PT samples. Herein, we highlight the technical and logistical limitations to this practical solution and promote the commercial development of lineage-specific cell subset chimerism testing PT samples.

Overall, this NGS-based MC assay showed robustness across the battery of proficiency testing performed as none of the samples required repetition due to insufficient yield post-preparation of the DNA library or insufficient DNA read numbers hindering interpretation. This is evident in the high concordance (99.9%) achieved in measuring mixed chimerism across a concentration of 0.3%–50% and the reproducibility testing series showing a nearly 100% concordance for inter-assay reproducibility on the same and multiple runs, amongst our technologists, interpretation across different software versions, with different NGS instruments and cell flow kits. Therefore, the NGS-based chimerism monitoring assay showed high precision across the aforementioned concentration range, indicating high assay consistency and reproducibility across various variables.

Our NGS-based assay’s analytical limit of detection is 0.3% and allows for extremely miniscule levels of host DNA detection, which is critical for early detection of relapse or donor failure. Contrastingly, the lowest reported limit of detection in the literature for STR-based MC monitoring is 1% (Kreyenberg et al., 2003; Schraml et al., 2003; Lion et al., 2012; Faraci et al., 2018). Additionally, the specificity of this NGS-based MC assay of 99.9% will enable accurate distinction of the donor and host DNA and thereby minimize the false-positive or false-negative rates. These analytical metrics can also be used to establish clinical guidance for the timing and intensity of immune modulation therapy, such as immune suppression or donor lymphocyte infusions. This NGS-based MC assay had the lowest limit of DNA input (1.25 ng) compared to other chimerism monitoring assays in the literature, such as variable-number tandem repeat PCR (100–250 ng), short tandem repeat PCR (1–5 ng), real-time quantitative PCR (20–300 ng), digital droplet PCR (20–100 ng), and other NGS assays (5–50 ng) (Sreenan et al., 1997; Lion et al., 2001; Alizadeh et al., 2002; Acquaviva et al., 2003; Chalandon et al., 2003; Thiede et al., 2004; Lassaletta et al., 2005; Karlen et al., 2007; George et al., 2013; Kim et al., 2014; Qin et al., 2014; Willasch et al., 2014; Stahl et al., 2015; Aloisio et al., 2016; Roloff et al., 2017; Waterhouse et al., 2017; Chen et al., 2018; Kim et al., 2018; Kliman et al., 2018; Mika et al., 2019; Pedini et al., 2019; Tyler et al., 2019; Valero-Garcia et al., 2019; Tripathi et al., 2020; Pettersson et al., 2021). Our assay’s low input DNA limit, high sensitivity, and high specificity make it suitable for implementation for accurate and reliable chimerism monitoring in our large allo-HSCT population. Furthermore, with the emergence of microtransplantation, our assay operating parameters will enable seamless integration of micro-chimerism monitoring.

A comprehensive understanding of the timing of lineage-specific cell-subset immune reconstitution post-allo-HSCT is crucial for discerning lineage-specific engraftment dynamics. The neutrophils reconstitute early (14–30 days), followed by NK cells (30–100 days), T cells (100 days), and finally, B cells (1–2 years) (Ogonek et al., 2016). The MC correlation was slightly higher in the CD3^+^ (0.959)-enriched samples compared to the unfractionated blood cells (0.946). Contrastingly, CD66^+^-enriched cells (0.535) did not attain a high correlation. Interestingly, the most common causes for engraftment failure are graft-versus host and its treatment (30–50%), relapse from premorbid hemato-oncologic disease (20–50%), and host T-cell-mediated rejection of donor HSC (Barrett and Battiwalla, 2010; Zeiser and Blazar, 2017). All these entities are driven by T cells, and therefore, CD3^+^-enriched cells showed a higher linear correlation when comparing NGS and STR. The literature shows that MC analysis with lineage-specific cell subsets has a higher sensitivity compared to unfractionated blood samples (Antin et al., 2001; Horn et al., 2009). This increased sensitivity bolsters chimerism analysis in lineage-specific cells when compared to unfractionated cells. Additionally, the fold change in MC detection for CD3-enriched compared to unfractionated input samples ranged as high as 38.9-fold, which would clinically translate to an earlier trigger for intervention. Furthermore, CD66^+^-enriched cells showed no congruent mixed chimerism compared to unfractionated or CD3^+^-enriched cells. Our dataset for CD66^+^-enriched cells showed a host % of nearly 100%. Since neutrophils reconstitute early post-allo-HSCT, we observe nearly 100% of these positively selected myeloid cells. Additionally, myeloid cells are not the predominant initiators of engraftment failure, and the CD66^+^ cell subset can be utilized to monitor the relapse of myeloid lineage malignancies.

Important technical aspects for the implementation of cell subset isolation and MC monitoring in allo-HSCT patient samples are the interplay between cell purity, cell isolate yield, and specimen processing time. We utilized a positive selection method via a cell lineage-specific isolation, leading to higher cell purity with a lower cell subset yield. Contrastingly, negative selection-based isolation of cell subsets may be advantageous in specimens with a larger number of unwanted cells compared to the target-enriched cells (Hanson et al., 2013). Therefore, cell purity and yield are dependent on the frequency of the enrichment target and total cell count. The amalgamation of cell subset isolation and high-sensitivity NGS-based chimerism monitoring capable of detecting MC necessitates a pressing need for standardization of cell purity cut-offs. Other technical considerations include the time and cost of specimen processing for lineage-specific chimerism testing. In our experiments, the additional cost for each lineage-specific cell subset enrichment was approximately 50 USD. Our automated cell sorter system has high-throughput capabilities as it can run multiple samples in the same run. This automated platform successfully isolated four cell subsets sequentially from the same whole blood sample within 2 h. Due to the reliable automated cell sorting, cross-contamination between samples was mitigated, which is critical to the success of downstream chimerism analyses. The high upstream cost for the automated instrument and the additional time for sample processing are compensated by the high sample purity, low cross-contamination, and reduced additional full-time equivalent laboratory staff required to upkeep large specimen volume demands.

The current Canadian gold standard for MC monitoring is an STR-based assay. This assay has many limitations such as the co-localization of peaks presenting as stutter peaks, susceptibility to preferential amplification, labor-intensive assay setup, and data analysis requiring specialized expertise. Furthermore, a large proportion of allo-HSCT donor–recipient pairs tend to be related, making many of the loci non-informative. The STR-based MC monitoring dataset in our study showed that the average number of informative loci in unrelated (6 ± 0.0) and related (5.2 ± 0.19) donors was separated by a single locus. Conversely, our NGS-based MC assay has a larger distinction between the informative alleles within the unrelated (120.10 ± 1.61) and related (72.35 ± 2.45) donors. This wider range of informative loci can also detect incorrect pre-transplant genomic samples as it compares and creates a genetic profile of the samples by assessing genome similarity and relatedness, and identifying any discrepancies between the expected donor and recipient profiles. Although pre-transplant recipient and donor genome profiles serve as references in most MC monitoring assays, the NGS-based MC assay can be performed in the absence of either the pre-transplant recipient or donor samples as the software application can extrapolate based on any one of the reference genomes. Additionally, NGS is also adaptable when the host has multiple donor transplants, a situation we often encounter in our transplant service. All these factors favor NGS-based assays over STR-based assays for the implementation of MC monitoring in moderate to high volume allo-HSCT centers.

A recent web-based survey by Blouin et al. revealed that lineage-specific chimerism testing was as high as 70% (n = 38) in the respondent laboratory (Blouin et al., 2021). This study showed that most laboratories used lineage-specific cell subsets with CD3-positive T cells (68%, n = 37 laboratories), CD33/CD66B-positive myeloid cells (52%, n = 28 laboratories), and CD19-positive B cells (28%, n = 15 laboratories) (Blouin et al., 2021). Additional candidates for cell subsets included CD56/CD16-positive NK cells (22%, n = 12 laboratories), CD34-positive hematopoietic stem cells (18%, n = 10 laboratories), CD14-positive cells (9%, n = 5 laboratories), and CD71-positive erythroid precursors (n = 1 laboratory) (Blouin et al., 2021). Despite this traction in utilizing lineage-specific cell sorting for MC monitoring, there is no known commercially available PT provider offering proficiency testing for MC in cell subset isolates. Currently, PT is performed on unfractionated blood samples in these laboratories, which do not truly represent the laboratory’s performance metrics regarding cell isolation yield, purity, and chimerism detection. Other logistical and technical limitations in the implementation of PT testing for cell subset isolates will be the increase in cost of obtaining commercially developed PT samples, the additional blood volume requirement to attain an adequate yield, and the urgency in shipping these samples at room temperature to avoid cell surface immunomarker loss, which will affect cell isolation. To circumvent these limitations, we utilized a proxy PT for cell subset MC testing in the form of an interprovincial comparative study between our NGS-based assay and the current Canadian gold standard assay (STR-based MC assay).

One of the limitations to this study is that all these unfractionated blood samples or cell subset isolates were collected in patients within their 1st year post-allo-HSCT at various time points. The next iteration of this study will aim to collect these samples at 1 month, 3 months, 6 months, and 12 months post-allo-HSCT to assess the temporal MC profile of lineage-specific cell subsets. In the absence of commercially available proficiency testing of chimerism in lineage-specific subset isolates, we can utilize inter-laboratory comparison with our study partner at the Cancer Care Manitoba HLA Laboratory and lean on our external quality assessment (EQA) programs via ASHI.

In conclusion, chimerism monitoring by NGS on cell subset isolates is highly accurate compared to STR-based assays and can provide early triggers for intervention through early detection of relapse and microchimerism. Despite the additional cost and time allocation, incorporating cell subsets and developing PT can increase the reliability of lineage-specific cell subset MC monitoring. The utilization of lineage-specific cell subset NGS-based MC testing in a medium-to-high volume allo-HSCT center is justified by the shorter turn-around time. Lastly, the pre-analytical advantages of utilizing low DNA input and the freedom to conduct multiple runs on both recipient and donor genomic samples make NGS-based MC monitoring assays reign supreme.

## Data Availability

The datasets presented in this study can be found in online repositories. The names of the repository/repositories and accession number(s) can be found in the article/Supplementary Material.
