# Supplementary material for: Validation of next-generation sequencing-based chimerism testing for accurate detection and monitoring of engraftment in hematopoietic stem cell transplantation
Source: Front Genet. 2023 Oct 23;14:1282947. doi: 10.3389/fgene.2023.1282947 (PMC10626454; doi:10.3389/fgene.2023.1282947)
Supplement: Supplementary file 1 [file DataSheet1.DOCX]

***Supplementary Sheet:* Validation of Next-Generation Sequencing (NGS) Based Chimerism Testing for Accurate Detection and Monitoring of Engraftment in Hematopoietic Stem Cell Transplantation**

**Supplementary tables**

**Table S1:** Summary of dilutions for linearity testing. Seven dilution mixtures were utilized as inputs for the NGS assay.

| **Dilution Series** | **DNA 1-Host (uL)** | **DNA 2-Donor (uL)** |
| --- | --- | --- |
| **Pure recipient** | **100** | **0** |
| **Pure donor** | **0** | **100** |
| **Mix 1: 0.3%** | **0.3** | **99.7** |
| **Mix 2: 0.5%** | **0.5** | **99.5** |
| **Mix 3: 1.0%** | **1** | **99** |
| **Mix 4: 3.0%** | **3** | **97** |
| **Mix 5: 5.0%** | **5** | **95** |
| **Mix 6: 10.0%** | **10** | **90** |
| **Mix 7: 50.0%** | **50** | **50** |

**Table S2:** Summary of input and output samples utilized to calculate the analytical specificity.

| **Sample** | **Recipient**  **=Ref1** | **Donor =Ref2** | **Recipient**  **= post-transplant** | **Donor**  **= post-transplant** | **Donor =Ref2** | **Recipient= Ref1** |
| --- | --- | --- | --- | --- | --- | --- |
| **Recipient** | 99.95% |  | 99.95% |  | 0.05% |  |
| **Donor** |  | 99.94% |  | 99.96% |  | 0.04% |

**Table S3:** Independent sample Mann-Whitney U test summary for comparison of informative markers in related and unrelated donor-recipient pair run on the NGS and STR based chimerism monitoring assay.

| **Chimerism monitoring assay** | **NGS** | | **STR** | |
| --- | --- | --- | --- | --- |
| **Donor-recipient pairs** | **Related** | **Unrelated** | **Related** | **Unrelated** |
| **N** | 20 | 39 | 20 | 39 |
| **Mean** | 72.35 | 120.1026 | 5.2 | 6 |
| **Std. Error of Mean** | 0.54904 | 1.61128 | 0.18638 | 0 |
| **Mann-Whitney U Test** | 780 | | 604.5 | |
| **Wilcoxon W Test** | 1560 | | 1384 | |
| **Standardized Test Statistic** | 6.267 | | 5.064 | |
| **Asymptotic Significance**  **(2-sided test)** | <0.001 | | <0.001 | |

**Table S4**: Comparison of the mean donor % on our NGS based MC monitoring and on the STR based MC monitoring with ASHI proficiency testing samples (n=15). ASHI approved target ranges for reportable results, good results and acceptable results are also summarized.

| **Our Lab (n=15)** | **ASHI Proficiency Testing samples (n=15)** | | | |
| --- | --- | --- | --- | --- |
| **NGS**  **(Mean donor %)** | **STR**  **(Mean donor %)** | **Reported Range** | **Good results**  **(Mean ± 2SD)** | **Acceptable Results**  **(Mean ± 3SD)** |
| **14.05%** | **14.58%** | 8-18% | 11-19% | 9-20% |
| **48.82%** | **48.41%** | 42-54% | 43-53% | 41-56% |
| **34.38%** | **33.75%** | 26-49% | 29-39% | 27-41% |
| **100%** | **100.00%** | 100-100% | 100-100% | 100-100% |
| **89.76%** | **89.16%** | 86-92% | 86-92% | 84-94% |
| **95.45%** | **94.47%** | 90-100% | 91-98% | 90-99% |
| **85.46%** | **84.94%** | 82-89% | 81-89% | 80-90% |
| **5.05%** | **5.32%** | 0-8% | 3-8% | 2-9% |
| **50%** | **50.32%** | 18-54% | 47-54% | 45-56% |
| **14.87%** | **15.12%** | 12-51% | 12-18% | 11-19% |
| **9.99%** | **10.58%** | 7-38% | 8-14% | 6-15% |
| **2.89%** | **3.19%** | 0-56% | 1-6% | 1-7% |
| **90.29%** | **89.79%** | 83-95% | 86-93% | 84-95% |
| **75.34%** | **74.54%** | 68-90% | 69-80% | 67-82% |
| **59.35%** | **59.32%** | 50-70% | 54-65% | 52-67% |
